# Detecting Sorghum Plant and Head Features from Multispectral UAV Imagery

**DOI:** 10.34133/2021/9874650

**Published:** 2021-10-01

**Authors:** Yan Zhao, Bangyou Zheng, Scott C. Chapman, Kenneth Laws, Barbara George-Jaeggli, Graeme L. Hammer, David R. Jordan, Andries B. Potgieter

**Affiliations:** ^1^The University of Queensland, Queensland Alliance for Agriculture and Food Innovation, Centre for Crop Science, Gatton, Queensland 4343, Australia; ^2^CSIRO Agriculture and Food, St. Lucia, Queensland 4072, Australia; ^3^The University of Queensland, School of Agriculture and Food Sciences, St. Lucia, Queensland 4072, Australia; ^4^Department of Agriculture and Fisheries, Agri-Science Queensland, Warwick, Queensland 4370, Australia

## Abstract

In plant breeding, unmanned aerial vehicles (UAVs) carrying multispectral cameras have demonstrated increasing utility for high-throughput phenotyping (HTP) to aid the interpretation of genotype and environment effects on morphological, biochemical, and physiological traits. A key constraint remains the reduced resolution and quality extracted from “stitched” mosaics generated from UAV missions across large areas. This can be addressed by generating high-quality reflectance data from a single nadir image per plot. In this study, a pipeline was developed to derive reflectance data from raw multispectral UAV images that preserve the original high spatial and spectral resolutions and to use these for phenotyping applications. Sequential steps involved (i) imagery calibration, (ii) spectral band alignment, (iii) backward calculation, (iv) plot segmentation, and (v) application. Each step was designed and optimised to estimate the number of plants and count sorghum heads within each breeding plot. Using a derived nadir image of each plot, the coefficients of determination were 0.90 and 0.86 for estimates of the number of sorghum plants and heads, respectively. Furthermore, the reflectance information acquired from the different spectral bands showed appreciably high discriminative ability for sorghum head colours (i.e., red and white). Deployment of this pipeline allowed accurate segmentation of crop organs at the canopy level across many diverse field plots with minimal training needed from machine learning approaches.

## 1. Introduction

The progressive increase in the global population and rising food consumption have placed unprecedented pressures on food security. There is also increasing demand to produce more food while reducing the footprint of agriculture on the environment [1, 2]. Over the years, breeding programs have played an important part in finding solutions to these challenges. In this regard, phenomics, especially phenotyping of traits, has evolved as a core selection tool of many breeding programs [3]. Phenotyping requires accurate and rapidly deployable quantitative metrics for determining physiological and morphological traits that can effectively assist the selection of elite varieties [4–8]. The selection of advanced varieties relies on the assessment of large numbers of diverse genotypes across multiple environments [9–12]. The quantification of crop-specific traits and interpretation of the physiological basis for genotype adaptation require pipelines for fast and accurate high-throughput phenotyping (HTP). This “bottleneck” remains a known impediment in implementing HTP in current breeding programs [13, 14].

The breeder's equation provides a framework that encapsulates the key factors involved in changing the rate of genetic progress that can be made in a breeding program. These factors include the amount of genetic variance, accuracy, and selection intensity, which in turn relate to population size and other crop phenotypic traits [15]. Determining such traits across large numbers of breeding lines through quantitative approaches with high speed and precision has always been paramount for breeding programs [16, 17]. Traditionally, traits such as plant density, stay-green, leaf angle, and lodging are manually measured through visual inspections at plot and field levels; however, these approaches are time-consuming, costly, prone to human error, and categorical in nature [18]. HTP methods are capable of improved accounting of the genetic variation across a large set of breeding lines, therefore enhancing selection efficacy and increasing the rate of genetic gain [15, 19]. In addition, during the past decade, phenotyping on-board ground- and aerial-based sensing platforms has been used in phenomics and has been shown to be highly successful in deriving specific traits relating to morphological, biochemical, and physiological functions at the canopy level [20–28].

Rapid development of consumer and enterprise UAV systems over the last decade demonstrates that such systems allow high temporal flexibility across dozens to hundreds of hectares of field area. Improvements in the accuracy of global position systems (GPSs) and in spatial and spectral resolutions of sensing units (e.g., multi- and hyperspectral cameras) further support the utility of UAV as the preferred technology for phenotyping, monitoring, and mapping crops across large areas [29–32]. Phenotyping using UAV-based cameras encompasses the derivation of sensing metrics that serve as surrogates for describing specific crop traits. These metrics rely on the fused properties of light as a result of the reflectance, transmission, and absorption of plant cells and organs at the canopy level and can be enhanced by the use of multispectral cameras [33].

In typical deployments of UAVs, thousands of overlapping images are captured and “stitched” into mosaics using robust software programs, like Agisoft [34] or Pix4D [35], that were originally based on the scale invariant feature transform (SIFT) algorithm [36]. After the alignment of overlapping images and processing (usually some form of pixel averaging), the product is a single geometrically corrected orthomosaic image that represents the instantaneous reflectance, which is affected by the integration of canopy structure, soil colour, and the bidirectional reflectance distribution function. The quality of an orthomosaic is affected during the rectification and pixel-averaging process, which limits its application [37, 38]. The orthomosaic is generated based on the digital surface model (DSM) that is created from the UAV-derived densified point cloud (DPC) and calibrated with a set of ground control points (GCPs) [35, 39]. A variety of reasons, for example, the lack of precision in GCPs, insufficient flight overlap, and movement of plant organs due to slight winds, would cause low accuracy of relative point heights and therefore errors in DPC. Such errors result in height differences in the recreation of canopy objects and lead to image distortions commonly seen in orthomosaics, especially for highly dense canopies and structural edges [35, 40]. This is also known as “ghost” or “halo effects,” which in crop-specific applications tend to blur the canopy features (e.g., leaves, flowers, and heads), thereby limiting its application for accurate phenotyping [37]. Methods for deriving high spatial resolution reflectance from original UAV photos without distortions are critical in quantifying canopy characteristics within the phenomics-genomics space.

While reverse calculation methods to derive plot images have been developed for UAV-based RGB photos for phenotyping purposes [40], few have been done to extract single images from a multispectral sensor onboard a UAV. In RGB photo-focused studies, high spatial information from plot images has been used for sorghum head detection using machine learning or deep learning algorithms [22, 41]. Geometric features of the heads (e.g., shape and size) have been applied to train mathematical models, which achieved moderate to high detection accuracies. Such studies generally require intensive labelling efforts for training purposes. Apart from the detailed spatial information used in these studies, the spectral information of the canopy has been less discussed. Recently, multispectral sensors have become more readily available for the development of practical applications in research [21]. The unique spectral characteristics of the canopy components provide additional properties that can be used for segmenting the components with fewer labelling and training efforts.

The aim of this study was to utilise the high spatial and spectral advantages of UAV-derived multispectral images for sorghum plant and head detection. An HTP approach was developed to determine the number of sorghum plants and heads at the plot level across large numbers of breeding plots. This was done by deriving calibrated high spatial resolution canopy reflectance data for each plot from thousands of multispectral UAV images. Specifically, this study is aimed at (i) enhancing the calibration of raw images without the loss of spatial and spectral details and (ii) developing an approach to automatically detect sorghum plants and heads in breeding plots.

## 2. Materials and Methods

### 2.1. Study Site, Imagery Collection, and Data Preparation

**Figure 1:**
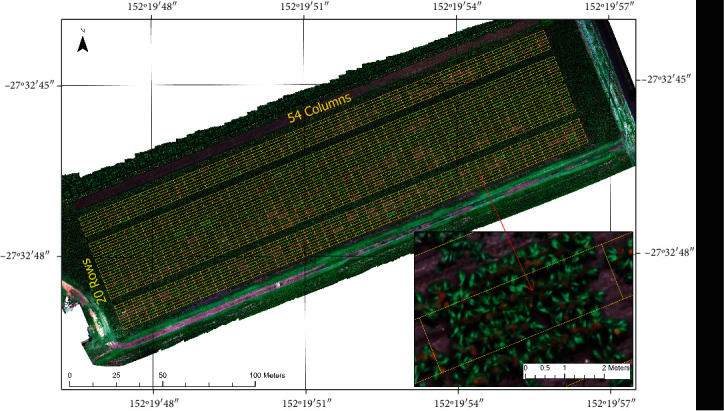
Layout of the sorghum breeding trial conducted in Queensland, Australia, during the 2019/20 summer season. The inset photo demonstrates the “ghost” effect associated with UAV orthomosaics.

This study was conducted at the experimental station of Gatton Campus, University of Queensland, during the 2019–2020 summer season (Figure 1). The trial was sown on 12 November 2019 with genotypes planted in plots comprising four rows 4 m in length. The internal two rows with 0.6 m row spacing were used for data collection. The row spacing between the two data rows and the outside rows was 0.75 m. A total of 1080 plots were sown in this trial, with a layout of 20 rows and 54 columns. The trial was nominally planted to be a pure stand, with approximately 30 plants per row. However, due to the great variation in genotypes and seed quality, the plots had a large amount of variability and ranged from about 5 to 30 plants per 4 m row length. Hence, the trial unintentionally provided a useful dataset for the study of plant emergence and head counts.

A three-dimensional robotics X8+ multirotor drone (Berkeley, California) mounted with an Altum narrow-band multispectral camera (MicaSense Inc., Seattle, Washington) was used to collect high-resolution and multispectral images. The Altum camera captures six bands: blue (475 nm centre, 32 nm bandwidth), green (560 nm centre, 27 nm bandwidth), red (668 nm centre, 16 nm bandwidth), red-edge (717 nm centre, 12 nm bandwidth), near-infrared (842 nm, 57 nm bandwidth), and thermal (not used here). The horizontal field of view (FoV) of the multispectral lenses was 48° with an 8 mm focal length, producing images at a resolution of 3.2 megapixels. Flight missions were conducted across the season on clear and cloudless days, and the flights were completed around mid-morning (10–11 am). The multispectral images were acquired from a height of 20 m.

To evaluate the performance of the developed methods, nadir images for 120 plots were manually labelled. LabelImg (https://github.com/tzutalin/labelImg) was used to create bounding boxes for sorghum heads (and plants) and to create ground truth data. The 120 plots were randomly selected, and for each plot, a nadir image (obtained by the method described in Section 3.2) was used to draw the bounding boxes. The flight data collected on 26 November 2019 (Table 1) were selected to label the presence of green plants, as this was the earliest flight for the season, and the recently emerged plants were small and not overlapping each other in most plots. The data collected on 19 February 2020 (Table 1) were selected to label the sorghum heads, as the sorghum heads were clearly visible and easy to identify. The selected images covered a wide range of different plant densities (from 3 to >100), different head sizes (e.g., small/medium/large), and different colours (e.g., red, brown, and white), which represented the variation across the entire breeding experiment.

When labelling, a rectangular bounding box was drawn surrounding each head (plant). After all heads (plants) in a plot were identified and labelled, the bounding boxes were cross-checked by another researcher. The number of heads (plants) was then automatically determined by counting the number of records in the LabelImg exported XML file.

### 2.2. The Pipeline and Its Applications

#### 2.2.1. An Overview of the Pipeline

**Figure 2:**
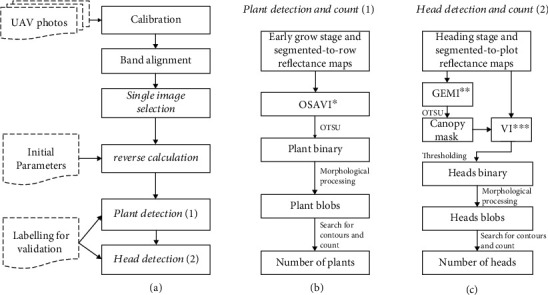
An overview of the pipeline depicting (a) the mainstream of the pipeline to obtain a nadir image for each plot, (b) the procedure for plant detection and counting, and (c) the procedure for head detection and counting (^∗^Optimised Soil Adjusted Vegetation Index, ^∗∗^Global Environmental Monitoring Index, and ^∗∗∗^Vegetation Index).

The pipeline (Figure 2) was designed to control the calibration workflow and overcome the defects associated with the software packages to generate orthomosaics for multispectral cameras. The nadir reflectance map for each plot was derived by following the procedures outlined in the mainstream of the pipeline (Figure 2(a)). The maps were then imported to the plant (Figure 2(b)) and head (Figure 2(c)) detection applications using the approaches specified in the following sections.

#### 2.2.2. Single Image Calibration for Surface Reflectance

The calibration model (Equation (1)) provided by MicaSense (https://support.micasense.com/) was implemented to calibrate the digital numbers (DNs) of the raw images into radiance:

(1) $$\begin{array}{c} L=V\left(x,y\right)\times \frac{a_{1}}{g}\times \frac{p-p_{\text{BL}}}{t_{\text{e}}+a_{2}y-a_{3}t_{\text{e}}y}, \end{array}$$

where *L* is the spectral radiance (W/m^2^/sr/nm), *p* is the normalised DN value, *p*_BL_ is the normalised black level value, *a*_1_, *a*_2_, and *a*_3_ are the radiometric calibration coefficients, *V*(*x*, *y*) is the vignette polynomial function for pixel location (*x*, *y*), *t*_e_ is the image exposure time, and *g* is the sensor gain settings. These model parameters were determined by accessing the metadata from the image metadata using the ExifTool (http://exiftool.org) in the pipeline.

The radiance (*L*) was further calibrated into reflectance by using the calibrated reference panel (CRP), which had been recorded prior to the flight. The known reflectance levels of the CRP (*ρ*_*i*_, provided by MicaSense) and the average value of radiance for the pixels located inside the panel area of the CRP image (avg(*L*_*i*_)) were used to determine the reflectance calibration factor (*F*_*i*_) for each band (Equation (2)). The factors were then used to convert all radiance values to reflectance for the images collected in the same flight.

(2) $$\begin{array}{c} F_{i}=\frac{\rho_{i}}{\text{avg}\left(L_{i}\right)}. \end{array}$$

#### 2.2.3. Spectral Band Alignment for Pixel Scale Calculation and Analysis

The MicaSense Altum possesses five lenses, excluding the thermal lens, that are not hardware aligned. To do pixel scale calculation and analysis, a software alignment must be applied to the spectral bands. The pipeline implemented an image alignment function based on motion models for this task [42]. In the analysis, a “Homograph” motion model, which accounted for the shift, rotation, and scale relationships between two image layers, was used to define the relative relationship between each pair of spectral bands. When implementing the model, the green band was set as the reference, and the other four bands were compared with the reference separately to generate a transformation matrix for each band. The bands were then brought into alignment by applying these matrices.

#### 2.2.4. Reverse Calculation for Segmenting the Plots

A research trial typically comprises a set of plots arranged in a matrix of rows and columns. This plot layout can be registered in ArcGIS and then applied to an orthomosaic. Reverse calculation is designed to use the orthomosaic and the plot layout to locate individual plots within the calibrated images and therefore segment the images for specific applications. The pipeline referred to the reverse calculation model developed by Duan et al. [40] and revised it for calibrated Altum images. The model required three parameters created by Pix4D when generating the orthomosaic: (1) the calibrated image position, (2) the transformation matrix (*P* matrix), and (3) the DSM.

In the reverse calculation for segmenting rows (for plant detection), plant rows (straight lines) were first digitalised in ArcGIS using the orthomosaic and buffered by 10 cm on both sides to create row boundaries. This was to avoid the weed plants distributed between the rows.

By overlapping the row boundary with the DSM, the 3D coordinates (*X*, *Y*, *Z*) of the boundary vertices in the coordinate system of the orthomosaic were calculated and subsequently converted to 2D coordinates (*u*, *v*) in the images given in pixels by applying the *P* matrix, according to the following equation:

(3) $$\begin{array}{c} {\left(x,y,z\right)}^{t}=P\text{ matrix}*{\left(X,Y,Z,1\right)}^{t},\\ u=\frac{x}{z},\\ v=\frac{y}{z}. \end{array}$$

The four (*u*, *v*) calculated coordinates were then used to generate a polygon within the image for segmenting the rows.

Since a plot could be captured by several images, it was essential to decide which image to use for the plot. In this study, the image selected for further analysis was the one in which the detected row was closest to the image centre (nadir). This was done by calculating the distance of the row centre to the image centre during the reverse calculation procedure.

Similar procedures were applied to plot boundaries for segmenting plots for head detection.

#### 2.2.5. Sorghum Plant Detection

Flight data collected on 26 November 2019 were selected for plant detection and counting (Figure 2(b)). The flight was 14 days after sowing, and the emerging plants were observable from the images with few plants clustered together in most of the plots. The OTSU automatic thresholding method was introduced to separate green pixels from the soil background. The OTSU method is an adaptive thresholding algorithm for binarization; it iterates all possible threshold values and returns an optimal threshold, minimising within-class variance [43]. The Optimised Soil Adjusted Vegetation Index (OSAVI) was selected for OTSU analysis after visually comparing the binarization results from other vegetation indices, including the normalised differential Vegetation Index and the Global Environmental Monitoring Index.

Morphological opening followed by morphological closing was then applied to the OTSU-generated binary image for each plot. The opening operation was to perform etching on the image first and then dilation, which would smooth the edge of the object without changing its contour and eliminate the small noise points generated during the masking procedure. The closing operation first performed a dilation and then etching to make up the holes. The number of green pixel clusters was then detected and counted with a blob detector searching individual pixel clusters within the plot [44].

#### 2.2.6. Sorghum Head Detection

Flight data collected on 19 February 2020 were selected for sorghum head detection when the sorghum heads in most plots were well established (Figure 2(c)). Two challenges exist in estimating the number of heads in the plots. The first was to separate the head pixel clusters from the complex background, including soil, green leaves, shadowed leaves, bright leaves, and heads. The second was to count the number of heads where single heads were mixed with combined heads (head pixel clusters containing two or three single heads).

**Figure 3:**
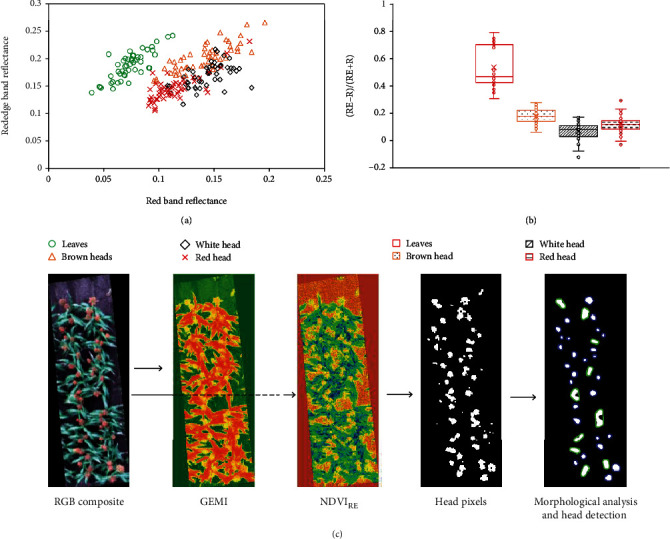
Separability of canopy elements. (a) The separability between heads and leaves using red and red-edge spectral bands. (b) The value range of the normalised differential Vegetation Index using red and red-edge bands for leaves and heads. (c) The use of GEMI and NDVI_RE_ indices for masking candidate sorghum head pixels.

A two-step threshold strategy was implemented to progressively mask the background information. The OTSU method was applied to the Global Environmental Monitoring Index (GEMI) to mask the soil background in the first step [45]. GEMI was compared and selected rather than OSAVI or NDVI due to its slightly better ability to separate the canopy (green leaves and heads) from the soil background without losing head pixels. A threshold of 0.3 was applied to a normalised differential Vegetation Index using red and red-edge bands (hereafter called NDVI_RE_) to separate head pixels from the rest of the canopy. The index and threshold were determined by examining the spectral information for canopy leaves and heads. As shown in Figure 3(a), the green leaves were well separated from head clusters by the red and red-edge bands. The threshold was then determined by checking the value ranges for green leaves and heads with different colours (Figure 3(b)).

After applying the GEMI OTSU and NDVI_RE_ thresholds, binary images mainly representing head clusters were generated (Figure 3(c)). Morphological opening and closing operations were then implemented to refine the binary images for the preparation of head detection and count. The position of sorghum heads was then determined by searching the bright pixel cluster and drawing contours for each cluster. The number of head clusters (single, double, or triple head cluster) was determined by the number of contours found in the plot.

**Figure 4:**
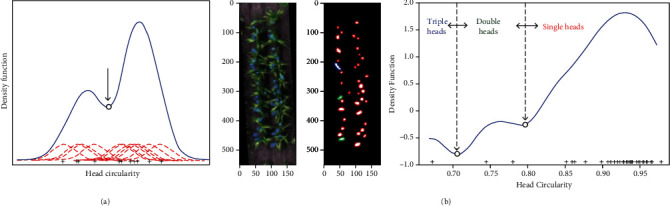
Separating single, double, and triple head groups using Kernel density method. (a) Demonstration of kernel density estimate. The red dashed curves are the six individual kernels (Gaussian), and the blue curve is the kernel density estimate. (b) The application of the Gaussian kernel density method for finding the threshold in separating single (red), double (green), and triple heads (blue) in the demonstration plot.

Circularity was selected as a shape descriptor to quantify the difference between the head clusters. Theoretically, a single near-circle sorghum head presents circularity close to one. When single heads overlap with adjacent heads, the circularity of the groups will decrease. A kernel density estimate scheme was employed to automatically determine the threshold(s) for separating the groups. To implement this, the circularity for each head cluster was calculated. A Gaussian kernel was then applied to each cluster, and the kernels were aggregated to generate the densify function. The local minimums were searched and taken as threshold values to separate the cluster into different groups (Figure 4). The total number of heads in each plot was then calculated as the ^“^number of single head clusters + the number of double head clusters × 2 + the number of triple head clusters × 3.^”^ Clusters with more than three heads were rare in this dataset.

#### 2.2.7. Validation and Analysis

To evaluate the performance of the pipeline, the derived sorghum plant and head maps were first intensively checked visually against the corresponding nadir image for the plots. Objects subjected to overdetection or missed detection were traced along the pipeline to check the possible causes. The pipeline-derived results for the 120 manually labelled plots were compared with the manual counts. Statistics including the coefficient of determination (*R*^2^) and root mean square errors (RMSEs) were calculated to evaluate the accuracy.

## 3. Results

### 3.1. Reverse Calculation for Plot Nadir Image

**Figure 5:**
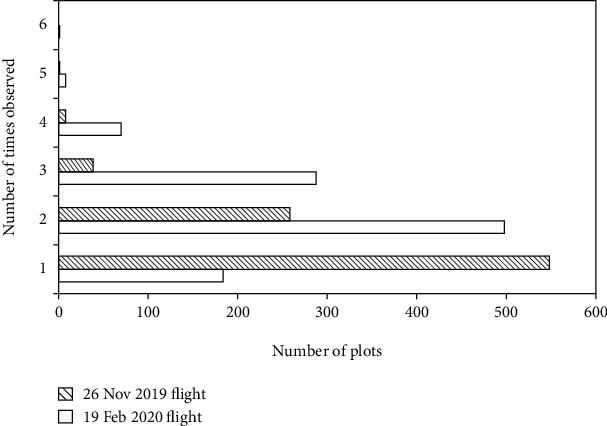
Distribution of observation times for plots that were fully observed by the calibrated images.

**Figure 6:**
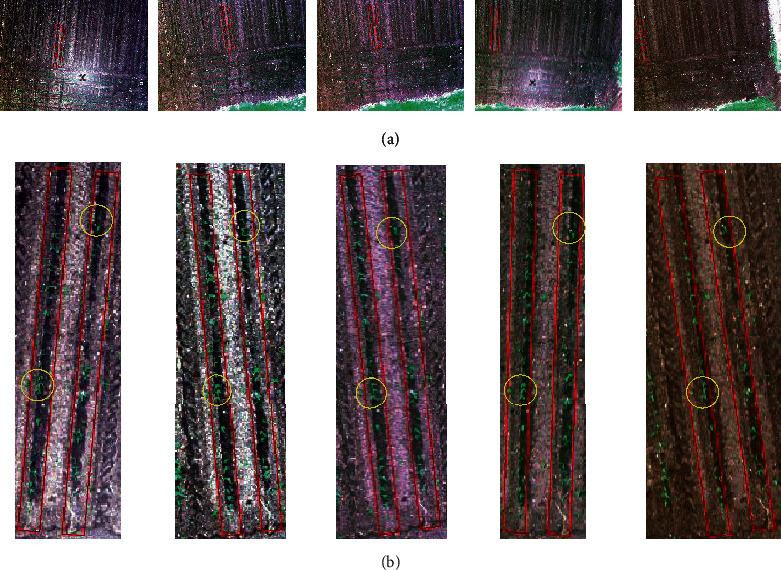
Reverse-calculated row images for plant detection. (a) One plot captured in five raw images and (b) the derived rows, with increasing distance to the image centre. The first image from the left was selected as nadir and was used for plant detection.

At the early vegetative stage (26 November 2019), a total of 1034 UAV image captures were collected across the trial, with 854 plots being completely captured. Most of the plots (548 plots, Figure 5) were completely captured in only one capture, and 258 plots were completely captured in two captures. Only one plot was captured in five captures (Figure 6). Due to the low flight altitude (20 m) and insufficient overlapping of images along and across the flight path, the remaining 226 plots were not fully captured in single images.

The reverse-calculated nadir image was selected for the plant detection test. As shown in Figure 6, the emerging plants in the nadir image presented fewer overlaps than the non-nadir images. The comparison also revealed that, as the position of the plot moved towards the margin of the images, the quality of the image decreased, and the plants became blurred. In addition, the reverse calculation-derived row boundaries also shifted for the plots located away from the image centres, which could be attributed to the increased distortions towards image margins.

**Figure 7:**
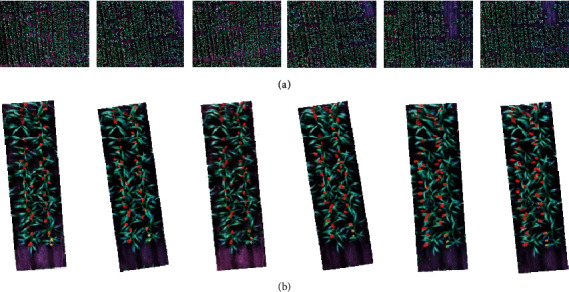
Reverse-calculated row images for head detection. (a) One plot captured in six raw images and (b) the derived plots, with increasing distance to the image centre.

Similarly, at the heading stage (19 February 2020), a total of 1588 UAV captures were collected across the trial, with 1049 plots completely captured in the images. Most of the plots (498 plots, Figure 5) were completely captured at least twice. One plot, shown in Figure 7, was captured six times. Due to increased capture overlap along the flight path (compared to the 26 November flight), only 31 plots were not fully captured.

The plot images that were reverse calculated from the calibrated images presented good quality overall, regardless of their distance to the image centre. However, significant differences in canopy and sorghum head characteristics were observed between the nadir and non-nadir images. Specifically, the nadir image viewed from the top of the plot presented an overall smaller canopy coverage when compared to non-nadir plot images (Figure 7). The sorghum heads from the nadir image showed a near-cycle shape and were relatively easy to distinguish when combined (e.g., head clusters with two or three heads). This was key to the fundamental method of using circularity to identify single and overlapping double and triple heads within the plots. However, the non-nadir images viewed from the side of the canopy showed elongated, eclipse shapes with substantial variations, which made it difficult to use a simple shape parameter for distinguishing head clusters and affected the results, as seen in Section 3.3.

### 3.2. Automated Detection of Sorghum Plants

**Figure 8:**
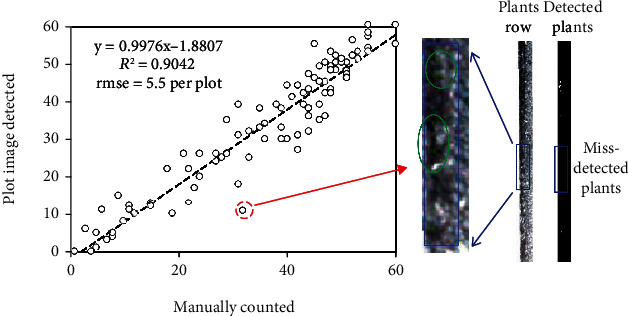
Accuracy of sorghum plant numbers determined by the reverse-calculated plot images compared with manually labelled images.

The number of green plants determined with the proposed method agreed well with the manually counted results, with the distribution of the scatterplots aligning relatively well with the 1 : 1 line (*R*^2^ = 0.90, Figure 8). However, some plots showed large uncertainties (e.g., the indicated dot in Figure 8). The main reason for this seems to be the failure to detect the extremely small emerging plants, which consisted of only a few pixels. It is possible that these few green pixels were masked during the thresholding procedure or eliminated during morphological operations.

**Figure 9:**
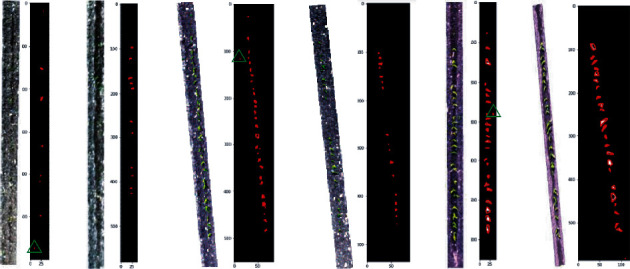
Examples of detecting and counting green sorghum plant results.

The plant detection examples shown in Figure 9 further demonstrate the performance of the proposed method. Overall, the method worked efficiently in detecting most green plants of moderate to large sizes with few overlaps. Some overestimations were observed mainly due to the weed plants distributed close to the sorghum plants (e.g., green plants located in the green triangles in Figure 9). Missed detections were also observed, which can be attributed to (1) failed detection of extremely small plants (Figure 8 and Figure 9) or (2) the overlapping of plants detected as one plant (Figure 9).

### 3.3. Automated Detection of Sorghum Heads

**Figure 10:**
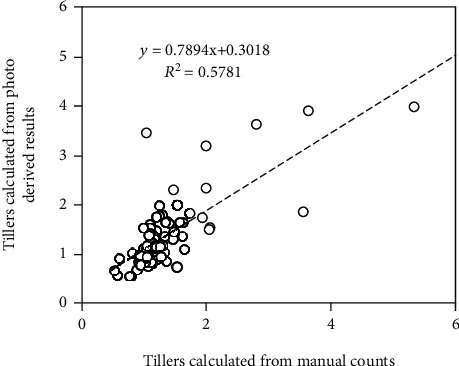
Predicted tiller numbers compared with observed data per plot.

**Figure 11:**
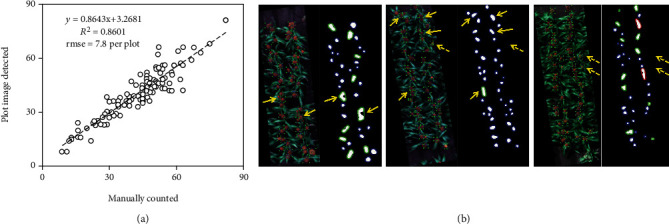
(a) Accuracy of sorghum heads determined by the reverse-calculated plot images compared with manually labelled images. (b) Detection results compared to labelled images. Solid yellow arrows show underestimated heads (i.e., triple heads identified as double in image set 1; double heads identified as single in set 2), and dashed arrows show missed detections of green heads in image set 3 (blue: single head; green: double head; red: triple head).

**Figure 12:**
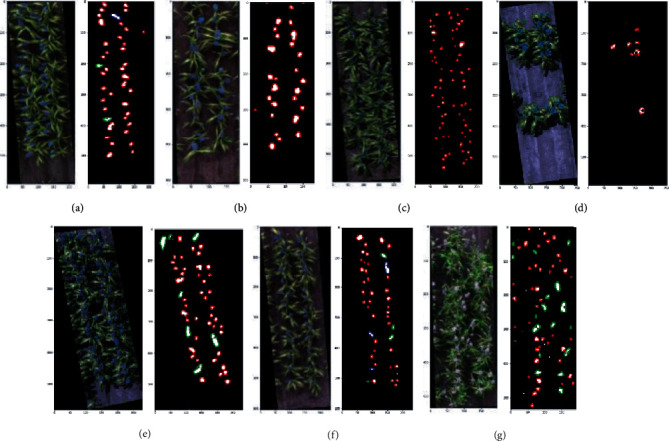
Examples showing sorghum head detection results for the plots; red contours indicate a single head, green contours indicate a double head, and blue indicate a triple head.

Detection was based on the spectral differences of head pixels from their complex backgrounds, and the proposed method worked relatively well in separating the head pixels. The detection results agreed well with the manually counted results, regardless of the size, density, and colour of the heads (*R*^2^ = 0.86, RMSE = 7.8 heads per plot, Figures 10 and 11(a)). The best detection results were achieved in plots with a moderate plant density and with genotypes with relatively large and compact heads (e.g., Figures 12(a), 12(b), and 12(e)). The selected shape parameter of circularity performed well in identifying the differences among single, double, and triple head clusters (e.g., Figures 12(e) and 12(f)). The method also successfully detected the head clusters in plots showing different head colours (e.g., white in Figure 12(g)). Relatively poor results were observed in plots with large but open heads (Figure 12(d)), where the thresholding procedure failed to generate complete pixel clusters. Only the connected portions of the head were kept during thresholding, and it became difficult to identify the heads using circularity. When investigating the head detection results against labelled plots, the number of heads was slightly underestimated compared to the observed number in some plots. Specifically, some triple heads were identified as double heads and some double heads as single heads. Green heads that had just appeared could not be clearly detected, which was mostly due to aligning the timing of the flight with the phenology stage (Figure 11(b)).

## 4. Discussion

In this study, an HTP pipeline was developed for detecting sorghum organ traits, i.e., the number of plants and heads. Specifically, this study (i) derived high spatial and spectral information from single UAV multispectral imagery and (ii) discriminated crop components from the surrounding background based on high-resolution spectral signals. This framework provides high accuracies similar to those of recent studies that focused on the implementation of pure machine learning algorithms but had less demand on labelling and computing resources.

### 4.1. Inversing Matrix of the Spatial and Spectral Attributes from Single UAV Imagery

The use of UAV-derived orthomosaics for HTP purposes is limited due to “ghost effects” surrounding plant canopies that are introduced during stitching procedures, which typically average the pixel values across slightly misaligned images [37, 40, 46]. This study derived the nadir images from calibrated multispectral UAV imagery, which could provide an efficient pipeline for HTP applications, including sorghum plant and head extractions. The derived approach had no coregistration with adjacent images, which is one of the main causes of “ghost effects” in orthomosaics, while keeping the high spatial and spectral resolutions of the original individual raw image [38, 47].

In addition, the transformation matrix utilised in this study generated the relative relationships between the band pairs using visible features across all bands and therefore required no additional reference information from ground measurements. Furthermore, the rich spectral information was reserved at each pixel by aligning the multispectral bands. Another advantage of this approach is that the transformation matrix shows some stability during a flight unless the UAV platform undergoes shock events [48]. Therefore, depending on the available computation resources, band alignment can be implemented either using a single transformation matrix applied to all flight captures or calculating individual matrices for each capture and implementing transverse alignments accordingly.

For the best results, flight heights, flight path overlaps, and flight times need to be carefully designed during flight scheduling. To implement the proposed framework for all plots across the field, it is important to ensure that each plot is completely captured in at least one image. For instance, in this study, in the flight data collected on 29 November 2019, only two-thirds of the plots had derived single plot images due to insufficient overlaps. For operationalising this approach, options to segment all plots are threefold: (i) to increase the flight frontal and side overlap, (ii) to increase the flight height so that the FoV covers larger areas with enough spatial resolution, and (iii) to add functionality to segment a plot from adjacent images in cases where the plots were not fully visible in one image. The number of plants in some of the plots in this study was also underestimated due to the relatively large size of plants in these plots and the plants being overlapped. An earlier flight might provide better plant detections for these plots. Similarly, for head detection, the method failed to count the plots with opening heads, which might be partially attributed to the time the images were captured.

### 4.2. Multispectral Data for Characterising Crop Traits

Previous research studies in the domain of remote sensing applications in vegetation inferred the importance of spectral information in discriminating between crop canopies and noncanopy and/or soil features [49, 50]. With this understanding, the proposed approach does not require extensive labelling of individual canopy components in each plot. This is different from the RGB UAV-based analysis [22] or deep learning- (DL-) focused methods [41]. Although the performance of these methods has dramatically improved, there remains a large labour and computing cost to achieve peak predictive performance. For example, the DL methods need extremely large training sets for training and thus increased accuracy. Such training sets are enormously time and labour intensive to create. Here, the developed method showed significant efficacy in (i) separating the canopy organs (e.g., green leaves and heads) without the need for including an extensive labelling approach. The significantly high agreement between the derived results from this framework and the manually counted number of sorghum plants and heads exemplified that the rich spectral information derived from the multispectral sensors provides sufficient skill in characterising plant organ-related traits.

In addition, this approach can also be adjusted to account for merged heads and/or plants, which allows for further out-scaling across variable organ types and colours. For example, in Ghosal et al. [41], images with white sorghum heads were not included in their training, which led to poor performance when applying their model to plots with white heads. The scalability of current DL feature extraction methods across a wide range of plant organ types remains a challenge and needs to include the full range of variations associated with the targeted plant traits [51]. In this study, the spectral signal of white sorghum heads still shows sufficient distinction from the green canopy to use a threshold to separate the two. The segmentation of head pixels was based on their unique spectral characteristics in comparison to soil and green canopy backgrounds. Its performance dropped when there was a significant number of green heads in the plots (Figure 11(b)).

Finally, the proposed approach does not require the adjustment of threshold values when applied to different plots with changing light and canopy densities. Thresholding methods to detect plant traits from the colour, shape, and size of the canopy features showed a significant ability to derive plant organs [22]. However, their performance was highly dependent on the selection of the thresholds, and the optimum threshold values for each plot differed due to light conditions, as well as differences in genotype by environment. The derived approach overcame manual thresholding by harnessing the unique spectral signal of canopies, thus allowing the application of a fully automated OTSU thresholding algorithm to select the optimum threshold for each single image inside each plot without any manual intervention [43, 52]. However, OTSU thresholding did not allow for a clear separation between weed areas and sorghum plants in some cases. Higher spatial and spectral information will be required to identify the subtle differences between the plants. Instead of manually buffering rows with known row widths (e.g., 20 cm), as done in this study, David et al. [53] developed an automated approach to accurately determine row locations and directions, which could be adapted for extracting precise cropping rows. This would increase the accuracy and scalability of the proposed framework.

### 4.3. Morphology Attributes for Detecting Overlapping Sorghum Heads

Accurately counting the number of heads when they overlap, due to pixel resolution during flight period, remains a challenge [20, 22]. Here, the circularity of the detected head blobs was used to successfully identify the presence of single and overlapping double and triple heads. Circularity was selected because it is a metric independent of the size of the heads. Other morphological metrics, such as area and perimeter, were further tested but showed poor accuracy. This was mainly due to attributes like area and perimeter of the head blobs changing both within a plot and across multiple plots [22]. This made it difficult to implement an automated thresholding algorithm for plant detection, while an arbitrarily determined threshold would fail in plots with significantly changed sorghum heads.

To overcome this, a Gaussian kernel density function was utilised to automatically determine the presence of overlapping heads in the plots. Traditionally, thresholds for identifying clusters are determined by examining the shape of the histogram. However, a major problem with histograms is that, depending on the number of bins, the choice of binning can have a disproportionate effect on the results. The Gaussian kernel density created a smooth density estimator as a powerful nonparametric distribution of the circularity levels within a plot. With continuous distribution, it was possible to find the thresholds for single, double, and triple heads by searching for the local minimums.

There are some limitations to this method. First, the highest number of overlapping heads considered in this study was set at three. For this study, after some inspection, the number of overlapping heads was limited to three, since higher numbers of overlapping heads were extremely rare and would reduce the overall accuracy. Second, circularity works well in identifying circular single heads and overlapping circular heads, which were observed in most nadir images where the heads had clear near-circular boundaries. However, it failed in cases where a nadir image was not available, and the heads were captured from an oblique angle. In this case, the heads presented an elongated shape and therefore decreased circularity, which might be identified as overlapping heads instead. In addition, the circularity parameter might have a lower ability in cases where heads do not correspond closely to a circular shape. Further research is needed to address this issue.

### 4.4. Implications for Crop Breeding Efforts and Limitations

Knowing the number of plants and heads in sorghum breeding plots is important for the selection of varieties that are higher yielding across different environments [12, 21, 23, 31, 32]. In this study, the proposed method showed good efficacy in detecting plant organs, thus counting sorghum plants and heads. Apart from that, the method showed an appreciably good ability to detect plant organs across a wide range of genotypes and emergence conditions and therefore is likely to provide breeders with better information and knowledge of crop adaptation across other environments and management practices.

Furthermore, the estimation of plant and head numbers also provides an estimate of tiller number and hence the propensity to tiller of a genotype, which is known to have a beneficial impact on yield [54, 55]. In these experiments, the tiller number per plant (approximated by the number of detected heads divided by the number of plants inside a plot) ranged between 0.5 and 2, which is well within the range for tillering reported elsewhere for sorghum [56]. In addition, the derived number of tillers per plant per plot agreed well with the calculations from manually counted plants and heads for the plots (*R*^2^ = 0.58). Our study exemplified the utility of a proximal sensing framework on board a UAV to derive not only plant and head counts but also tiller numbers at much faster turnaround times. It is also more cost-effective with appreciably higher accuracies compared to manually collected field data.

## 5. Conclusion

Using UAV imagery to characterise crop traits has become a focus in developing timely, accurate, and cost-effective phenotyping platforms. While approaches using RGB photos collected with UAVs have been commonly developed for crop plant and head detections, fewer studies have focused on the applications derived from multispectral imagery. The latter has the advantage of capturing additional spectral wavelength information, which can likely be utilised to enhance the ability to detect plant organs. However, to effectively harness the increased spectral range and information, a single image scale approach is required. Here, to detect the number of sorghum plants and heads from a multispectral camera, a semiautomated HTP pipeline that utilises a single image per plot was developed, thus preserving spatial and spectral data integrity. The approach showed a significant ability to align the spectral bands, calibrate the reflectance, and extract a singular nadir image for each plot through the implementation of a reverse calculation approach. The methods applied here performed appreciably well in separating green plants from the soil background and sorghum heads from the complex canopy backgrounds. The number of plants and heads counted from nadir images showed high prediction accuracies when compared with observed data. Coefficients of determination were 0.90 and 0.86 for plants and heads, respectively. Finally, the proposed HTP framework developed here showed reasonable specificity for a wide range of plant densities, head sizes, and head colours. It is envisaged that this approach can be applied rapidly and cost effectively across many sorghum breeding plots, resulting in accurate information of crop responses to different environments for a wide range of genotypes.

## Figures and Tables

**Table 1:** Flight information for the postemergent stage and heading stage.

	26 Nov 2019	19 Feb 2020
Flight time	10:15–11:15 am	10:50–11:50 am
Flight height	~20 m	~20 m
Number of images collected	6204	9528
Number of captures	1034	1588

## Data Availability

Trial details, sample imagery, and essential codes used in this article can be accessed through https://github.com/YanZhao15/AltumApplication.git.
